# Temporal Waveform Reconstruction Algorithm for Fast Super-resolution Ultrasound Imaging

**DOI:** 10.34133/bmef.0314

**Published:** 2026-11-13

**Authors:** Yuhang Yang, Shunyao Luan, Runlang Zhu, Kailiang Xu, Benpeng Zhu

**Affiliations:** ^1^ School of Integrated Circuits, Huazhong University of Science and Technology, Wuhan 430074, China.; ^2^ Department of Radiation Oncology, Hubei Cancer Hospital, TongJi Medical College, Huazhong University of Science and Technology, Wuhan 430074, China.; ^3^ Ophthalmology Center, Renmin Hospital of Wuhan University, Wuhan 430060, China.; ^4^ School of Information Science and Technology, Fudan University, Shanghai 200438, China.

## Abstract

**Objective:** A processing framework is introduced to address robustness and computational challenges in translating ultrasound localization microscopy (ULM), enabling fast and adaptive microvascular imaging. **Impact Statement:** Parameter-free filtering and accelerated localization triple processing speed provide a reliable foundation for real-time clinical super-resolution microvascular imaging. **Introduction:** ULM surpasses the acoustic diffraction limit to resolve microvasculature. However, its clinical adoption is hindered by parameter-sensitive filtering and computationally heavy localization and tracking. **Methods:** First, a parameter-free temporal waveform reconstruction filter adaptively recovers microbubble temporal signatures from raw time-series data, separating them from tissue clutter and operating 5.7 times faster than conventional filters. Second, a Gaussian fit radial symmetry method accelerates localization by adding a Gaussian similarity step to the standard local maximum radial symmetry approach, reducing processing time to one-third. **Results:** The workflow processes each frame in 0.0065 s, down from 0.02 s per frame, achieving a 3-fold speedup. It delivers 10-μm-scale super-resolution microvascular imaging validated on multiple public datasets. **Conclusion:** This efficient and adaptive framework markedly cuts ULM processing time, paving the way to real-time clinical microvascular imaging.

## Introduction

Microvascular imaging plays a critical role in understanding the pathophysiology of conditions such as coronary heart disease [1], diabetes [2], and cancer [3]. However, current clinical imaging modalities for vasculature have significant limitations. For example, while micro-computed tomography provides high spatial resolution, its reliance on heavy metal contrast agents and exposure to ionizing radiation restrict its clinical use [4,5]. In contrast, optical coherence tomography is excellent for retinal imaging but offers limited penetration depth, making it unsuitable for deep-tissue microvascular assessment [6]. Furthermore, although widely available, ultrasound-based color Doppler flow imaging (CDFI) is fundamentally limited by the acoustic diffraction barrier, preventing the visualization of micron-scale vessels [7].

Ultrasound localization microscopy (ULM) has emerged as a technique that addresses these limitations. By precisely localizing and tracking intravenously injected microbubble contrast agents, ULM enables noninvasive, super-resolution imaging of the microvasculature in vivo [8,9]. Unfortunately, its clinical translation is hindered by 2 core challenges in the processing workflow: parameter sensitivity and prolonged computation times [10–12].

The first challenge lies in clutter filtering. The prevalent approach, which combines singular value decomposition (SVD) with band-pass filtering (BPF), exploits differences in spatiotemporal coherence between tissue and microbubbles [13]. Nevertheless, its performance depends on empirically determined thresholds. Recent advances aim to reduce this dependency. For instance, adaptive threshold selection via spatial similarity matrices has been proposed [14], and machine learning approaches, such as 3-dimensional convolutional neural networks, can replicate SVD filtering with less manual tuning [15]. Unsupervised models such as robust principal component analysis directly separate sparse microbubble signals [11], while semi-supervised frameworks (e.g., CS-Net) improve generalizability with limited labeled data [16]. Although these methods reduce reliance on manual parameters, their increased model complexity often raises computational costs, which conflicts with the need for real-time processing.

The second bottleneck occurs during localization. Traditional Gaussian fitting is computationally intensive due to its exhaustive search and iterative fitting per candidate [17,18]. Efficiency improvements often replace global search with local maximum detection and use faster estimators such as the centroid method [19]. Despite reducing per-frame processing time, these changes remain insufficient to meet the speed requirements of real-time clinical imaging for large, high-resolution datasets.

In response to these limitations, this work develops an integrated set of algorithms. The temporal waveform reconstruction (TWR) method is proposed to reduce reliance on empirical parameters in clutter filtering. TWR identifies the unique temporal signature of individual microbubbles at each sampling point, thereby isolating microbubble signals from tissue noise in the time domain. This physically grounded strategy not only eliminates parameter dependency but also increases filtering speed by 5.7 times. To address the localization bottleneck, the Gaussian fit radial symmetry (GF-RS) method is introduced. This method uses Gaussian fitting and center-point intensity screening to expedite microbubble detection, reducing the time required for localization and tracking to one-third while preserving all relevant information.

This integrated framework reduces dependence on empirical parameters while accelerating microvascular image reconstruction from 0.02 to 0.0065 s per frame, achieving a 3-fold speedup and enabling super-resolution microvascular imaging with 10 μm resolution. This work provides an efficient and practical route to real-time clinical ULM imaging.

## Results

Data are acquired on a Verasonics Vantage 256 multichannel ultrasound research platform, followed by ULM imaging of rat cerebral vasculature after contrast agent injection. To further validate the proposed algorithm, we also use a publicly available dataset [19] containing multiple in vivo animal models: rat brain, rat kidney, and mouse tumor.

The ULM processing pipeline in this study consists of the following steps. The TWR method is applied to raw ultrasound data for clutter filtering, replacing the conventional SVD approach. This step suppresses strong echoes from static or slowly moving tissues and enhances microbubble contrast. After filtering, microbubbles are localized by detecting local intensity maxima. The conventional local maximum radial symmetry (LM-RS) method for microbubble screening is optimized into a variant denoted as GF-RS, and a radial symmetry algorithm then accurately extracts the centroid coordinates of each microbubble. Based on these coordinates, cross-frame trajectory tracking links the same microbubble across successive frames using spatial proximity, thereby reconstructing complete motion trajectories. Finally, super-resolution reconstruction aggregates all localization points from the tracked trajectories to generate a high-resolution microvascular image that substantially exceeds the diffraction limit.

To evaluate the practical impact of different methods on super-resolution imaging outcomes, we apply the above pipeline to reconstruct the rat brain microvascular network using the conventional algorithm along with the 2 methods we propose. A systematic comparison of processing efficiency and image quality is presented in Table 1 and Fig. 1, among which, M.1 represents the combination of SVD and BPF for filtering, and LM-RS for positioning; M.2 represents the combination of SVD and BPF for filtering, and GF-RS for positioning; M.3 represents TWR for filtering, and GF-RS for positioning.

**Table 1.** Processing times of different localization methods for 600 consecutive image frames

Algorithm	Filtering time	Localization time	Tracking time	Total process
M.1	2.75 s	6.49 s	2.42 s	12.48 s
M.2	2.75 s	1.66 s	1.12 s	6.37 s
M.3	0.48 s	1.63 s	0.93 s	3.91 s

**Fig. 1.**
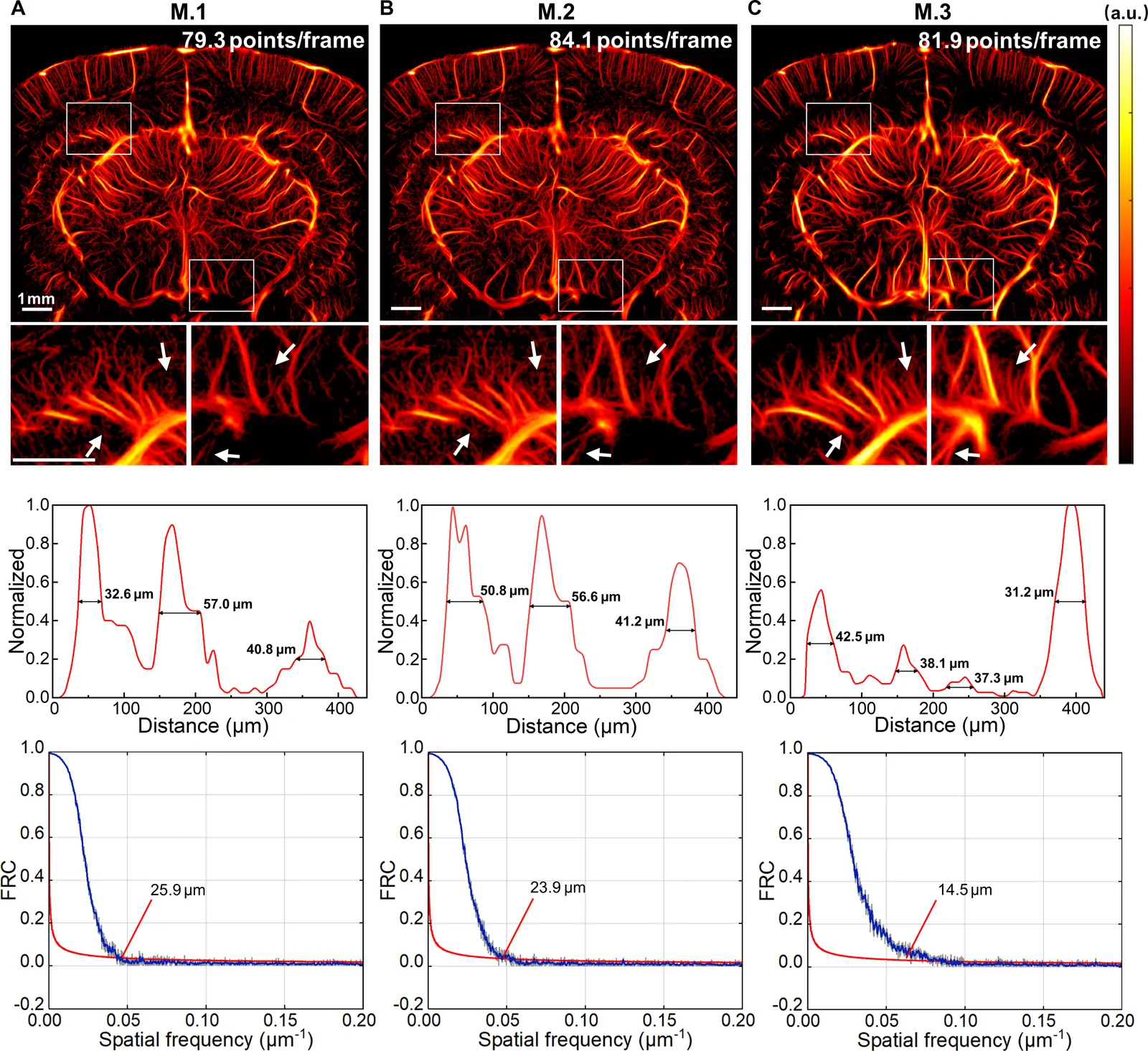
Super-resolution imaging of the rat brain using different processing methods: (A) M.1: SVD and BPF for clutter filtering with LM-RS for localization; (B) M.2: SVD and BPF for clutter filtering with GF-RS for localization; and (C) M.3: TWR for clutter filtering with GF-RS for localization. Each panel shows the super-resolution image, magnified regions, FWHM measurements along the white dashed lines, and the corresponding FRC curve. M.3 achieves superior imaging quality and vascular resolution.

### The GF-RS approach for microbubble screening

The performance of different microbubble screening methods is compared in Fig. 2. The conventional LM-RS and the proposed GF-RS localization methods are denoted as M1 and M2, respectively. To evaluate their performance, we use a noisy image frame as a test case and compare the microbubble points identified by the 2 methods. As illustrated in Fig. 2A, GF-RS captures prominent microbubble features while filtering out false signals induced by tissue artifacts, thereby yielding more accurate localization.

**Fig. 2.**
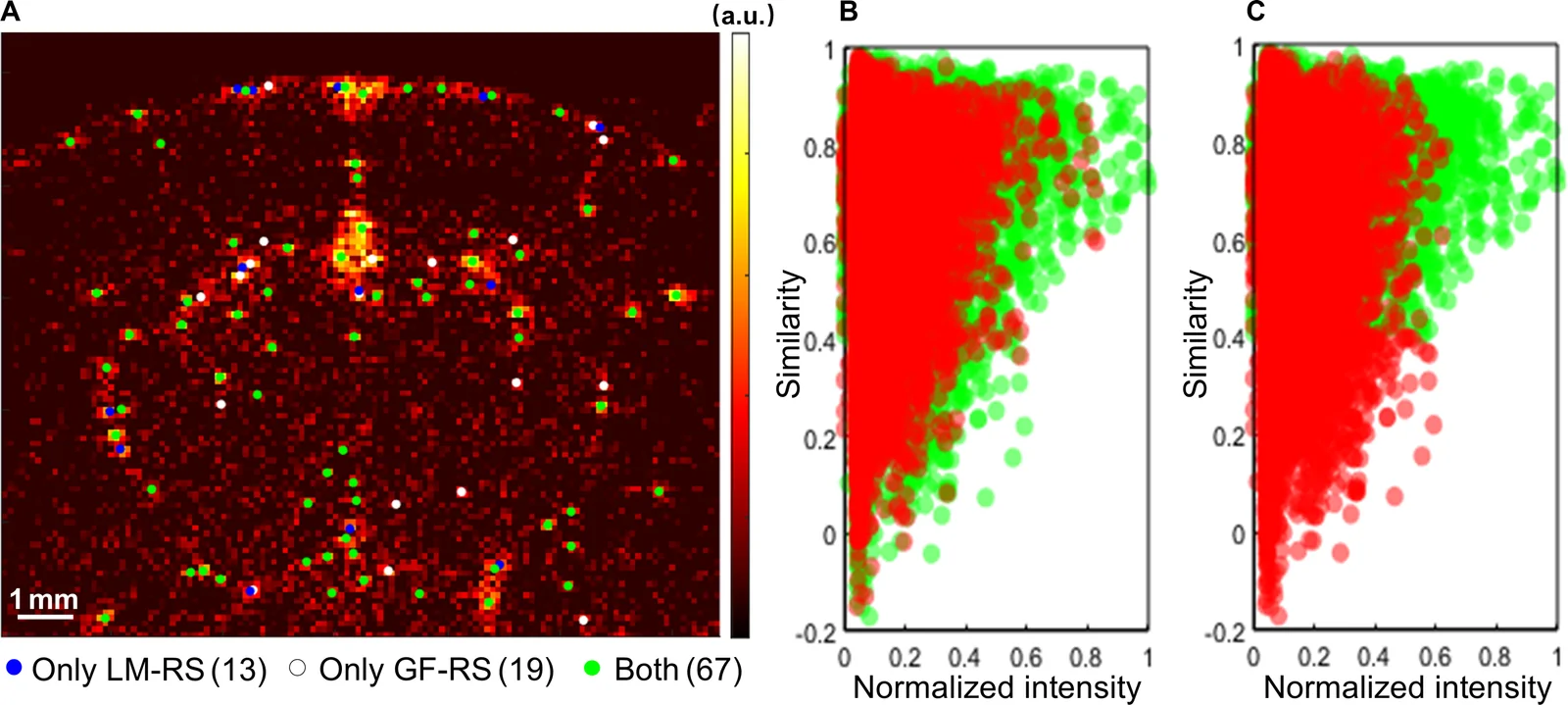
Comparison of localization and similarity between the LM-RS and GF-RS methods. (A) Detected points on a single frame. Green: matched points; white and blue: mismatched points. (B and C) Similarity distributions relative to an ideal Gaussian source for the same acquisition after filtering with (B) LM-RS and (C) GF-RS. Green: selected points; red: unselected points. GF-RS achieves more accurate microbubble localization than LM-RS.

Processing times are summarized in Table 1. The conventional method requires 6.49 s per frame, whereas GF-RS takes only 1.66 s per frame, achieving nearly 4-fold acceleration in screening and localization. The Gaussian similarity scatter plots reveal a clear distinction: candidate points from GF-RS have similarity values above 0.4, whereas those from the conventional method (which relies on local statistical features) are markedly more dispersed, with many points showing low similarity. This indicates that GF-RS more reliably identifies true microbubble signals that follow a Gaussian diffusion model. Furthermore, GF-RS doubles the speed of microbubble tracking without compromising imaging quality. This improvement stems from the intrinsic Gaussian-like spatial intensity distribution of real microbubbles. By leveraging Gaussian similarity, GF-RS better recognizes genuine signals, increases the matching rate during tracking, and thereby enhances overall tracking performance.

### TWR algorithm for clutter filtering

The single-frame filtering quality of the proposed TWR method is compared with that of conventional approaches using rat brain ultrasound data. As shown in Fig. 3, TWR markedly outperforms conventional methods in both clutter suppression and preservation of characteristic microbubble speckles. Compared with pipelines that rely on spatial-filtering-based preprocessing, TWR markedly improves filtering and denoising quality in ULM. In mouse brain imaging, we quantify resolution improvement with 2 complementary metrics. The Fourier ring correlation at a 2σ threshold [20] gives a global measure of localization precision. By this metric, TWR brings the overall resolution down from 25.9 to 14.5 μm. Full width at half maximum (FWHM) measures the local minimum resolvable diameter from vessel intensity profiles. It tells us more directly how sharp individual vascular structures appear in the image. Here, TWR yields consistently narrower vessel widths than conventional SVD-based filtering, and it picks out microvessels that SVD simply cannot distinguish. This matches what we see in Fig. 1B and C: TWR clearly separates adjacent microvessels that conventional methods leave smeared together.

**Fig. 3.**
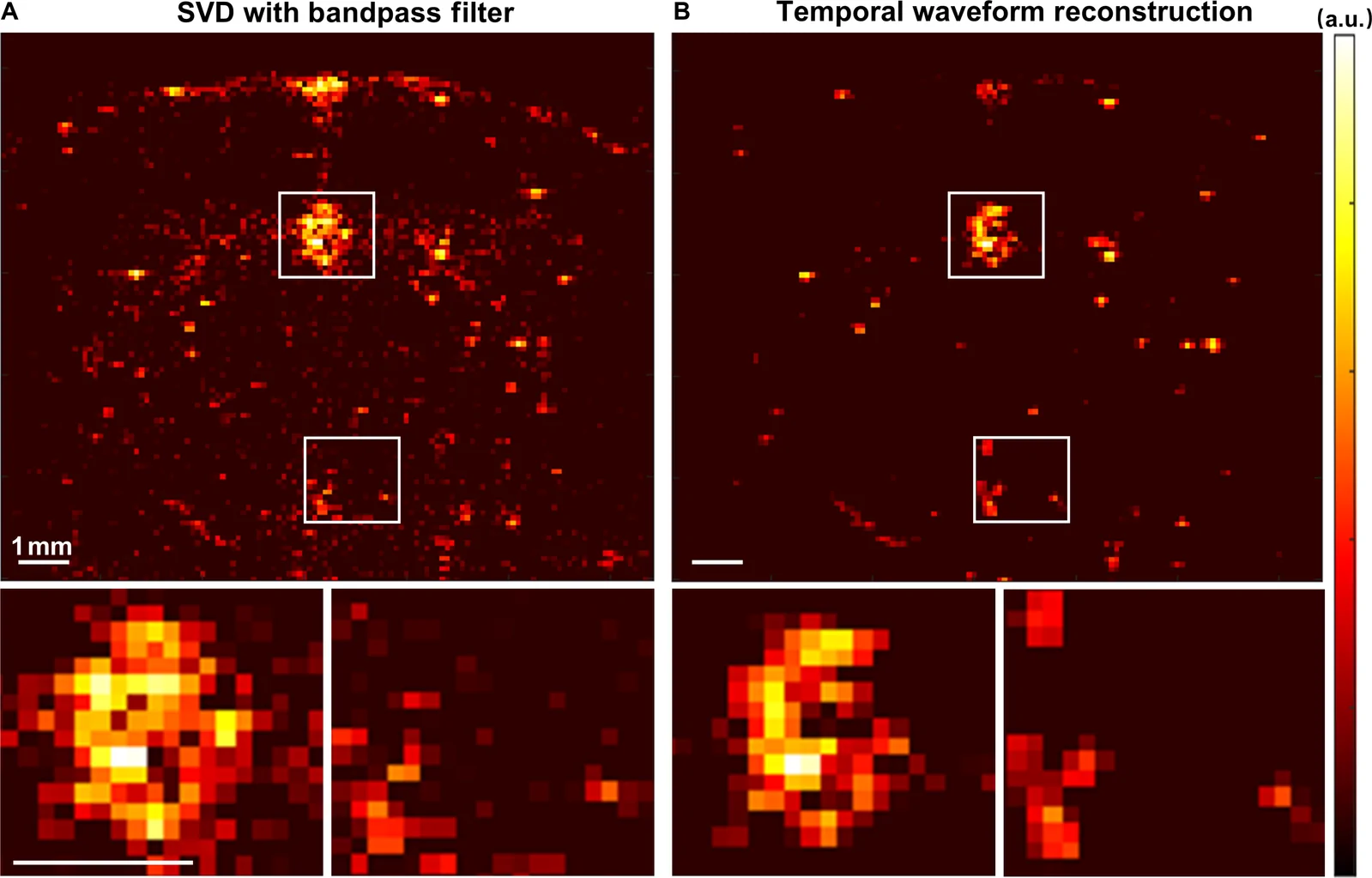
Filtered images of rat brain signals at a single time point, shown with corresponding magnified views. (A) Doppler images of ultrasound signals after SVD and bandpass filtering, alongside local enlargements. (B) Doppler images of ultrasound signals after TWR processing, alongside local enlargements. TWR suppresses tissue clutter more effectively while preserving microbubble signals.

By adaptively estimating the filtering window, the TWR method eliminates the need for empirical parameter adjustments depending on imaging site or flow velocity. It is also computationally efficient. These properties make TWR well-suited for real-time clinical applications.

### Validation on public datasets

We further validate TWR on public datasets covering rat brain, rat kidney, and mouse tumor, representing diverse physiological structures and hemodynamic conditions. Image resolutions from different processing methods are summarized in Figs. 4 and 5 and Table 2. Notably, TWR uses a consistent parameter-free workflow across all experiments, with no adjustments for organ or flow type. In rat brain imaging, TWR improves resolution from 17.7 to 10.6 μm and better resolves adjacent vessels. Across organs, TWR not only boosts processing efficiency but also refines microvascular structures via adaptive filtering. Under varied conditions, TWR consistently outperforms conventional filters in resolution and image quality. These results confirm the strong generalizability and robustness of TWR in complex clinical scenarios.

**Fig. 4.**
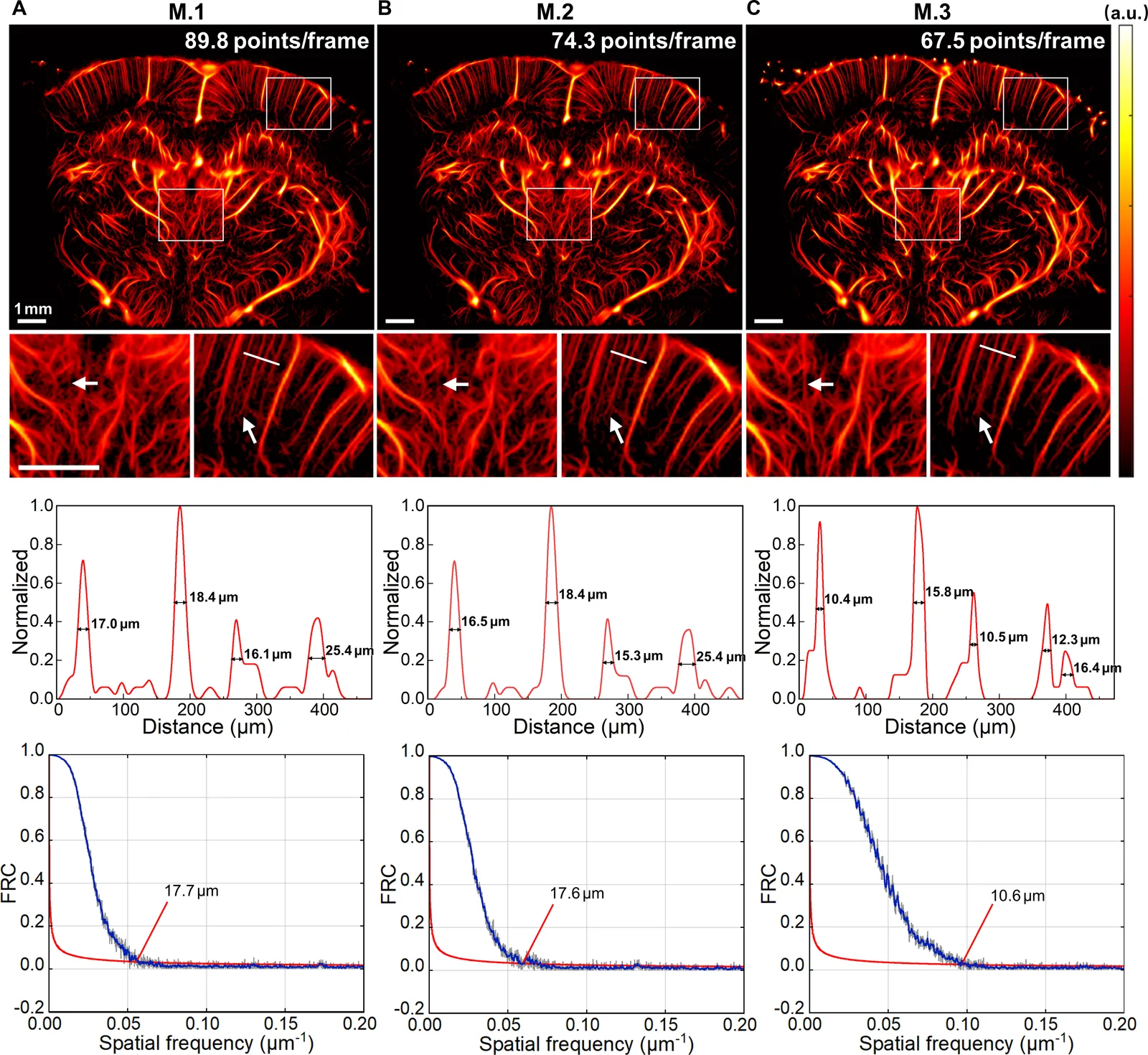
Super-resolution imaging of the rat brain from the public dataset using different processing methods: (A) M.1: SVD and BPF with LM-RS; (B) M.2: SVD and BPF with GF-RS; and (C) M.3: TWR with GF-RS. Each panel shows the super-resolution image, magnified regions, FWHM measurements along the white dashed lines, and the corresponding FRC curve. M.3 achieves superior imaging quality and vascular resolution.

**Fig. 5.**
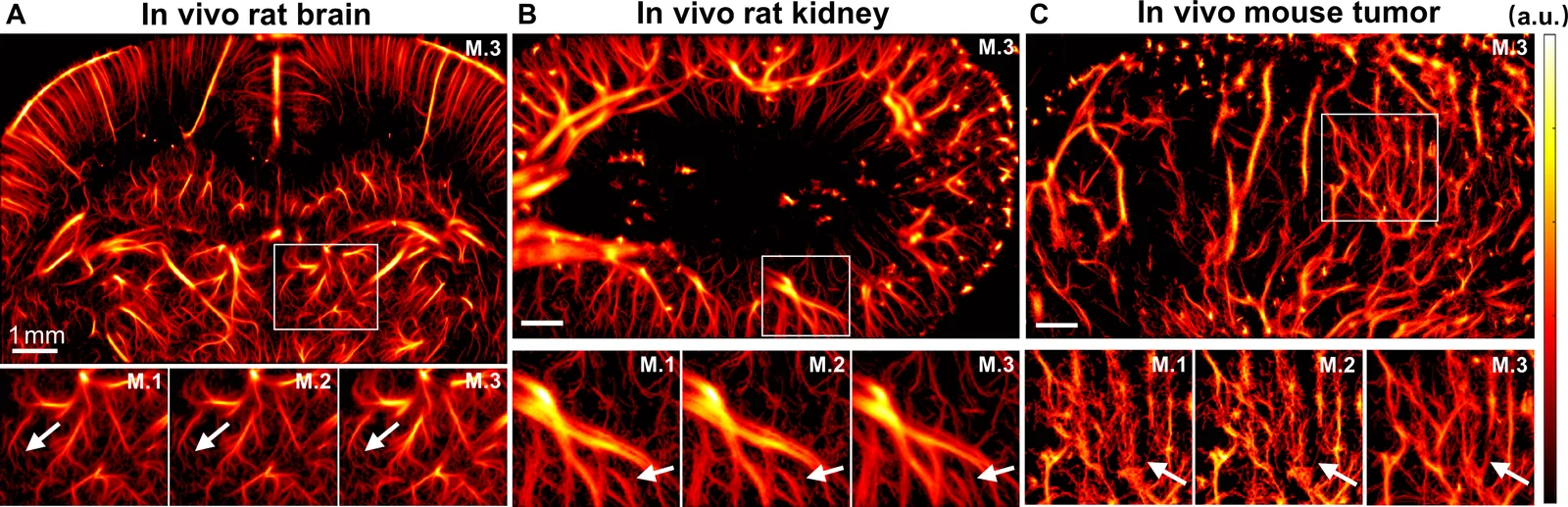
Super-resolution images and corresponding magnified views from public datasets: (A) in vivo rat brain, (B) in vivo rat kidney, and (C) in vivo mouse tumor. The proposed framework demonstrates good generalization performance across all three organs.

**Table 2.** Resolution of reconstructed images obtained with different processing methods

Algorithm	Brain bolus	Brain infusion	Kidney	Tumor
M.1	17.7 μm	12.6 μm	37 μm	35.8 μm
M.2	17.6 μm	10.7 μm	36.6 μm	37.2 μm
M.3	10.6 μm	10.8 μm	22.1 μm	16.6 μm

## Discussion

In this work, we propose an improved processing framework for ULM based on the TWR filter and GF-RS localization method. TWR effectively removes tissue clutter without empirical parameter tuning, and GF-RS substantially accelerates microbubble screening and localization. Together, these advances cut per-frame processing time from 0.02 to 0.0065 s and achieve super-resolution microvascular imaging at 10 μm resolution.

### Comparison with previous work

Conventional SVD-based filtering depends on manually selected truncation orders and cutoff frequencies [13]. Adaptive SVD [14] and machine learning methods [15,16] reduce parameter sensitivity but increase computational cost. In contrast, TWR automatically estimates the sliding window length from the data, requiring no user-defined parameters. With a speedup factor of 5.7 over conventional filters, TWR is well-suited for real-time applications. Few clutter filters combine parameter-free operation with high speed; TWR is one of them.

For microbubble localization, the proposed GF-RS method builds on the well-established LM-RS algorithm [19]. Whereas LM-RS relies on local intensity maxima and empirically set thresholds, GF-RS introduces a physics-informed Gaussian similarity metric. This modification improves robustness against noise and adjacent bubbles while cutting localization time by a factor of 3. Unlike deep-learning-based methods [18], GF-RS requires no training data or model optimization, making it more adaptable to varying imaging conditions.

### Implications and significance

This work facilitates the clinical translation of ULM by addressing key practical barriers. Real-time imaging is essential for intraoperative guidance and bedside diagnostics, and our framework’s overall 3-fold speedup brings ULM closer to that goal. TWR’s parameter-free design eliminates the need for site-specific recalibration, which is crucial for heterogeneous clinical data. Consistent performance across rat brain, kidney, and mouse tumor datasets underscores the generalizability of our methods.

From a broader perspective, the TWR filter introduces a new paradigm for separating microbubble signals from tissue clutter. It exploits the unique temporal waveform of flowing microbubbles rather than spatiotemporal coherence or frequency content. This insight could inspire further developments in time-domain signal processing for contrast-enhanced ultrasound.

### Limitations and future directions

Despite its advantages, the current implementation of TWR has several limitations. First, the method relies only on temporal amplitude information. In highly reflective regions, such as tissue–blood interfaces, residual edge fluctuations may produce local bright spots that slightly affect image uniformity, but this does not compromise the integrity of microvascular structures. Second, automatic window length estimation assumes a stable microbubble transit time. Extreme variations in flow velocity across vessel types can result in suboptimal window selection. Third, TWR has not been validated in organs with rapid, large-amplitude motion, such as the beating heart, where tissue displacement breaks the assumption of a stationary background within the sliding window.

For GF-RS, the use of a fixed Gaussian template assumes an ideal point spread function, which may vary with imaging depth and acoustic settings. While normalized cross-correlation (NCC) is robust against minor mismatches, severe aberrations could still reduce screening accuracy.

Several directions could address these limitations. Future work will add frequency-domain features and temporal continuity constraints to microbubble trajectory tracking to suppress edge-induced artifacts. For highly mobile organs, incorporating motion compensation strategies, such as dynamic-programming-based cross-correlation search used in recent studies [10], may be needed to extend TWR to cardiac imaging. Furthermore, incorporating spatial information into TWR enhances the recovery of spatial morphology while simultaneously improving the differentiation between microbubble signals and noise. Additionally, adaptive template generation based on local point spread function estimation could improve the robustness of GF-RS. Finally, we plan to implement GPU acceleration for the entire framework to achieve real-time processing on clinical ultrasound systems. Extending the system to 3-dimensional applications is also one of the future research directions.

In summary, this study provides an efficient, adaptive, and generalizable processing framework for ULM. The TWR filter and GF-RS method address 2 major bottlenecks: parameter sensitivity and computational intensity, while maintaining high image quality. With further refinements, this approach could accelerate the clinical adoption of super-resolution ultrasound microvessel imaging.

## Materials and Methods

### Experimental and technical design

This study presents a fast, adaptive signal processing framework for ULM that overcomes 2 major bottlenecks: parameter-sensitive filtering and computationally intensive localization. The workflow has 3 stages: TWR-based clutter filtering, GF-RS-based microbubble localization, and cross-frame tracking with super-resolution reconstruction. We validate the methods on in-house rat brain data and public datasets (rat brain, rat kidney, and mouse tumor), assessing processing time, microvessel FWHM, and spatial resolution. Detailed experiments are described in the corresponding subsections.

All processing was performed on a laptop with an Intel i5-10200H (2.40 GHz, 4 cores), 16.0 GB RAM, and an NVIDIA GTX 1650 Ti (4 GB), running Windows 64-bit. Algorithms were implemented in MATLAB R2023b with parallel acceleration.

### Animals and study approval

To comprehensively validate the robustness of algorithm, our study employs a combination of rat brain datasets that we acquired and 4 public benchmark datasets [19], covering rat brain perfusion, bolus injection, kidney, and mouse tumor, respectively. The brain dataset we acquired was collected using a Verasonics Vantage 256 system equipped with a 128-element L22-14vX linear array transducer (15.6 MHz). A multiangle plane-wave sequence dedicated to ULM was applied, with 5 tilted angles spanning from −5° to +5° and a pulse repetition frequency (PRF) of 5.0 kHz. Following GPU-accelerated delay-and-sum beamforming, the compounded frame rate reached 1,000 Hz, with each acquisition lasting 0.4 to 1 s. The 4 public datasets were also acquired with a 15-MHz center frequency (Vermon transducer, 0.1 mm pitch), but their parameters were adjusted according to the distinct physiological requirements of each organ. Specifically, the brain perfusion and bolus datasets adopted 3 angles (PRF 3.0 kHz, compounded frame rate 1,000 Hz) to capture fast cortical flow, whereas the kidney and tumor datasets employed 5 angles (PRF 5.0 and 2.5 kHz, compounded frame rates 1,000 and 500 Hz, respectively) to enhance clutter suppression. The signal-to-noise ratios of these public datasets range from 25 to 29 dB, and they encompass varying degrees of motion artifacts, from minimal (fixed brain) to moderate respiratory interference (kidney and tumor). On the basis of similar center frequencies, our acquired ULM data share a PRF of 5.0 kHz and a compounded frame rate of 1,000 Hz, consistent with the kidney dataset yet higher than the tumor dataset (2.5 kHz). This complementary configuration provides a robust testbed for evaluating algorithmic generalizability across diverse acquisition protocols.

The detailed parameters mentioned above (total frame counts, acquisition durations, and specific tilt angle ranges) are fully documented in the Methods sections of the original references and are therefore not reiterated here [19,21]. For a complete parameter comparison, readers are referred to the supplementary materials provided with those references.

### TWR filter

Temporal filtering approaches have been used in CDFI, typically involving the separation of orthogonal in-phase and quadrature (IQ) signal components to detect phase shifts associated with blood flow [22]. Yet, these methods are susceptible to misclassifying high-amplitude signals with subtle phase variations as flow under strong tissue interference. Furthermore, they can distort the spatial signature of microbubbles, which hinders their application in ULM. To address these limitations, the TWR filter is proposed. This method recovers the distinct temporal signatures generated when a single microbubble passes through a spatial sampling point, enabling effective separation from tissue noise (Fig. 6).

**Fig. 6.**
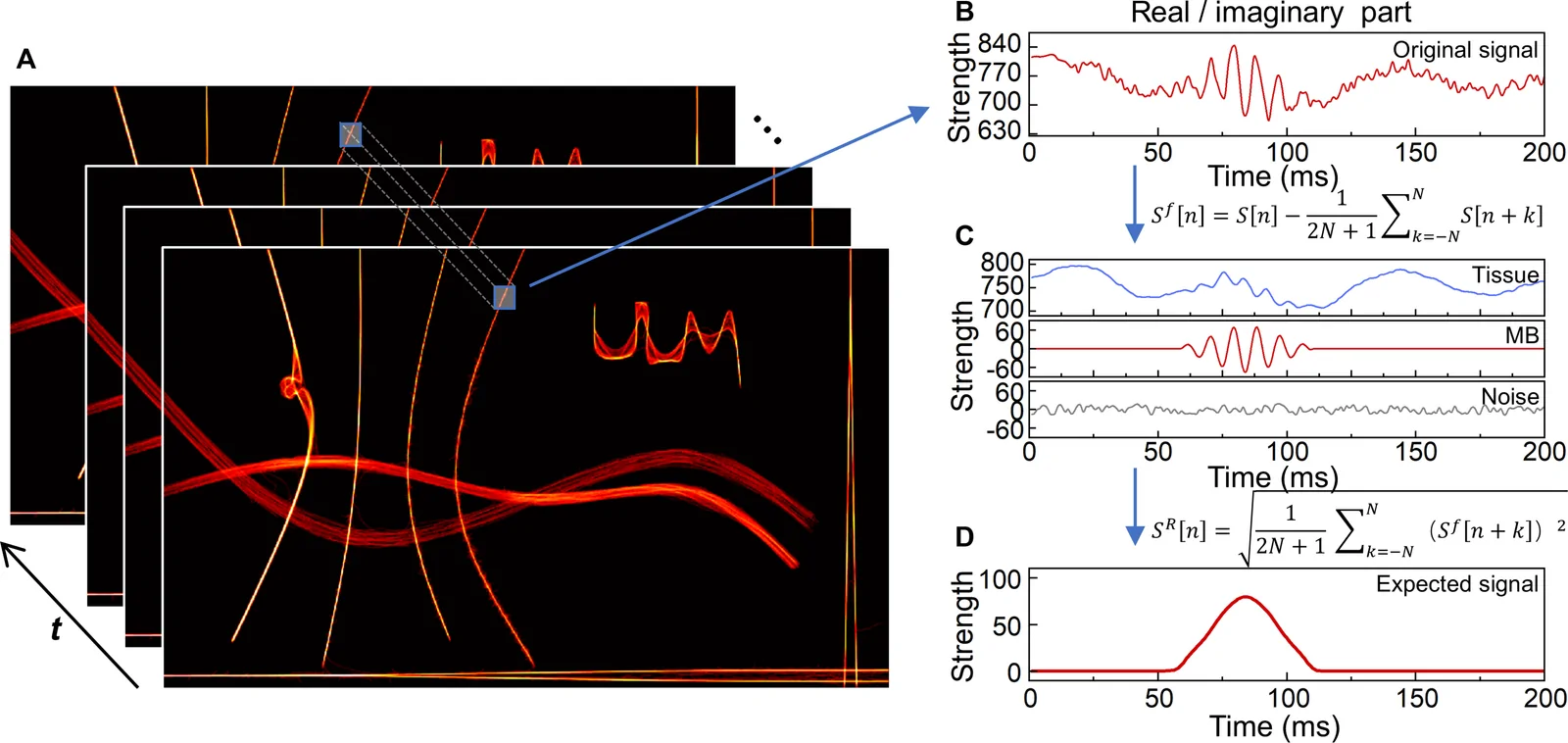
The TWR method. (A) Temporal signal at a sampling point. (B) Temporal evolution of the real/imaginary part. (C) Filtering to extract the pure microbubble echo. (D) Reconstruction into a broad-pulse signal for microbubble localization.

The oscillatory, nonlinear behavior of microbubbles produces broadband high-frequency echoes, whereas static or slow-moving tissue returns low-frequency fundamental signals [19]. To exploit this difference, the TWR filter applies a moving average separately to the real and imaginary IQ components along the temporal dimension, estimating the low-frequency tissue background. Subtracting this background from the original signals extracts transient microbubble components, providing initial clutter suppression and noise reduction (Fig. 6B and C). For a single spatial point, the operation is defined as:

$$\begin{array}{c} S^{\;f}\left[n\right]=S\left[n\right]-\frac{1}{2N+1}\sum_{k=-N}^{N}S\left[n+k\right], \end{array}$$ (1)

where S and $S^{\;f}$ are the original and filtered 1D temporal signals, and np=2N+1 is the sliding window length.

Although the filtered oscillatory signal contains essential harmonic information, it is not directly suitable for standard localization algorithms, which typically assume a smooth, single-peak profile. We therefore apply local average energy reconstruction to the residual signal. Specifically, we compute the instantaneous signal power, smooth it within a local temporal window, and convert it back to amplitude. This generates a smooth, wide-pulse signal that accentuates the microbubble’s energy footprint, as shown in Fig. 6C and D:

$$S^{R}\left[n\right]=\sqrt{\frac{1}{2N+1}\sum_{k=-N}^{N}{\left(S^{f}\left[n+k\right]\right)}^{2}},$$ (2)

where $S^{R}$ is the reconstructed temporal signal. The window length np can be set consistently for Eqs. (1) and (2).

The sliding window length *np* is automatically determined by the data. Specifically, take any frame of data for high pass filtering and calculate the dominant period corresponding to the residual blood flow component. Then, based on the period exported from the data, the window length *np* is set to ensure adaptive matching with local signal features.

### GF-RS localization

To improve the efficiency and accuracy of the localization and screening steps, the GF-RS method is employed. Given that microbubbles resemble a 2D Gaussian intensity distribution, we use the NCC coefficient to compare a candidate region of interest (ROI) with a Gaussian template, yielding a physics-informed, noise-robust measure:

$$\begin{array}{c} \begin{array}{c} \text{NC}C_{i}=\frac{\left[\sum_{x,y}\left(R_{i}\left(x,y\right)-\mu_{\text{Ri}}\right)\left(G\left(x,y\right)-\mu_{G}\right)\right]}{\left[\sqrt{\sum_{\left(x,y\right)}{\left(R_{i}\left(x,y\right)-\mu_{\text{Ri}}\right)}^{2}}\sqrt{\sum_{\left(x,y\right)}{\left(G\left(x,y\right)-\mu_{G}\right)}^{2}}\right]}, \end{array} \end{array}$$ (3)

where $\mu_{\text{Ri}}\;$ and $\mu_{G}\;$ are the mean pixel intensities of the ROI and the template, respectively.

Additionally, the method excludes ROIs whose intensity maxima are off-center, reducing localization bias caused by noise or adjacent microbubbles. This ensures that the detected center more accurately reflects the true microbubble position and enhances screening robustness. For computational efficiency, we implement the screening procedure with vectorized operations, eliminating slow pointwise iteration.

By improving filtering and localization, we accelerated the ULM pipeline to 0.0065 s per frame, a 3.1-fold acceleration over the conventional method (0.02 s per frame). Spatial resolution of super-resolved images also doubled, enabling 10-μm-scale microvascular imaging on multiple public datasets. These results offer a solid foundation for real-time clinical ULM.

## Data Availability

The data that support the findings of this study are available upon reasonable request from the authors.
